# Intra- and inter-operator variability in MRI-based manual segmentation of HCC lesions and its impact on dosimetry

**DOI:** 10.1186/s40658-022-00515-6

**Published:** 2022-12-21

**Authors:** Elise C. Covert, Kellen Fitzpatrick, Justin Mikell, Ravi K. Kaza, John D. Millet, Daniel Barkmeier, Joseph Gemmete, Jared Christensen, Matthew J. Schipper, Yuni K. Dewaraja

**Affiliations:** 1 Department of Biostatistics, University of Michigan, Ann Arbor, MI USA; 2 Department of Radiology, University of Michigan, 1301 Catherine, 2276 Medical Science I/5610, Ann Arbor, MI 48109 USA; 3 Department of Radiation Oncology, University of Michigan, Ann Arbor, MI USA; 4 Department of Radiology, UT Southwestern Medical Center, Dallas, TX USA

**Keywords:** Uncertainty analysis, Dosimetry, Segmentation, Radioembolization, Observer studies, Radionuclide therapy

## Abstract

**Purpose:**

The aim was to quantify inter- and intra-observer variability in manually delineated hepatocellular carcinoma (HCC) lesion contours and the resulting impact on radioembolization (RE) dosimetry.

**Methods:**

Ten patients with HCC lesions treated with Y-90 RE and imaged with post-therapy Y-90 PET/CT were selected for retrospective analysis. Three radiologists contoured 20 lesions manually on baseline multiphase contrast-enhanced MRIs, and two of the radiologists re-contoured at two additional sessions. Contours were transferred to co-registered PET/CT-based Y-90 dose maps. Volume-dependent recovery coefficients were applied for partial volume correction (PVC) when reporting mean absorbed dose. To understand how uncertainty varies with tumor size, we fit power models regressing relative uncertainty in volume and in mean absorbed dose on contour volume. Finally, we determined effects of segmentation uncertainty on tumor control probability (TCP), as calculated using logistic models developed in a previous RE study.

**Results:**

The average lesion volume ranged from 1.8 to 194.5 mL, and the mean absorbed dose ranged from 23.4 to 1629.0 Gy. The mean inter-observer Dice coefficient for lesion contours was significantly less than the mean intra-observer Dice coefficient (0.79 vs. 0.85, *p* < 0.001). Uncertainty in segmented volume, as measured by the Coefficient of Variation (CV), ranged from 4.2 to 34.7% with an average of 17.2%. The CV in mean absorbed dose had an average value of 5.4% (range 1.2–13.1%) without PVC while it was 15.1% (range 1.5–55.2%) with PVC. Using the fitted models for uncertainty as a function of volume on our prior data, the mean change in TCP due to segmentation uncertainty alone was estimated as 16.2% (maximum 48.5%).

**Conclusions:**

Though we find relatively high inter- and intra-observer reliability overall, uncertainty in tumor contouring propagates into non-negligible uncertainty in dose metrics and outcome prediction for individual cases that should be considered in dosimetry-guided treatment.

**Supplementary Information:**

The online version contains supplementary material available at 10.1186/s40658-022-00515-6.

## Introduction

There is much recent interest in dosimetry-guided personalization of radionuclide therapy with the goal of maximizing tumoricidal effect while limiting impact on normal tissue to an acceptable level [1–3]. Accurate and reliable patient-specific dosimetry is vital to achieving this goal, but can be challenging, especially for lesions.

Dosimetry is a multi-step process, and uncertainties are inherent in many of the steps, namely serial quantitative imaging and registration, volume of interest (VOI) definition, time–activity curve fitting and integration, and absorbed dose estimation [4, 5]. In the conventional MIRD-based approach, mean absorbed dose to a lesion is estimated by the product of the VOI time-integrated activity and a volume-dependent dose factor derived for a unit density sphere model [6]. Even when voxel-level dosimetry is performed by coupling patient images with direct Monte Carlo radiation transport for example, the lesion contour is applied to the dose map to derive mean absorbed dose [7]. Therefore, in both the conventional dosimetry approach and with voxel dosimetry, uncertainties in segmentation propagate to uncertainties in the lesion absorbed dose. Furthermore, partial volume correction (PVC) using volume-dependent recovery coefficients (RCs) is sometimes part of the activity quantification process for dosimetry, and is also affected by uncertainty in segmentation [4, 5].

Automated segmentation of select organs on anatomical imaging modalities using deep learning and atlas-based methods is now widely available. However, these automated methods are not yet sufficiently developed for segmentation of most tumor types because lesion size, shape, and location are highly variable, and tumor-to-normal-tissue contrast is often poor [8]. Tumor segmentation for dosimetry can be performed on emission images (SPECT or PET) or co-registered anatomical images (CT or MR). Automated count thresholding and gradient-based algorithms are often used for SPECT- and PET-based segmentation because of their speed and repeatability; however, due to the noise and limited spatial resolution of emission images, the resulting contours can have poor accuracy [9, 10]. Manual tumor segmentation on anatomic images exploits the high resolution of CT and MRI, but inter- and intra-observer variability inevitably exists, even when performed by imaging specialists [11, 12].

Gear et al. [4] and Finnochiaro et al. [13] investigated the uncertainty in each step of the SPECT-based dosimetry process and identified uncertainty in delineation of the VOI as the major factor. They derived an analytical equation that expresses volume uncertainty as a function of spatial resolution and voxel size for VOIs segmented on SPECT images by thresholding. This analytical approach is not suitable when using manual contouring on CT or MRI because other factors that do not enter into this equation, such as the impact of contrast, can dominate. An alternative approach to determining segmentation uncertainties and corresponding dose estimates is to perform a multi-operator study. Such multi-operator studies for manual lesion segmentation are rare in the internal dosimetry setting. To our knowledge, the one reported study by Meyers et al. [14] investigated inter- but not intra-operator variability. Furthermore, that study did not investigate variability associated with individual lesion contours, as the estimated quantity was the absorbed dose to the total tumoral liver and non-tumoral liver.

While multiple factors contribute to uncertainty in absorbed dose estimation, our study focuses on segmentation uncertainty, generally considered to be one of the main components. This is especially true in Yttrium-90 (Y-90) microsphere radioembolization (RE), a promising radionuclide therapy for hepatocellular carcinoma (HCC) and metastatic liver malignancies [3, 15]. In RE, intra-arterially delivered microspheres become trapped in the arterioles feeding the tumor and do not redistribute. Dosimetry can therefore be performed with a single PET or SPECT image of the activity distribution under the assumption that only physical decay contributes to the kinetics, thereby eliminating the uncertainty associated with co-registering serial emission images and time—activity curve fitting. We aim to quantify inter- and intra-observer variability in HCC lesion contours delineated manually on MRI images and the resulting impact on Y-90 PET/CT-based tumor dosimetry following RE. We also propagate the segmentation uncertainty to determine its effects on tumor control probability (TCP). We extended our study to perform a preliminary investigation of operator variability in measurement of lesion diameters, a parameter widely used to assess response in dose–response studies.

## Methods

### Imaging protocol

We used retrospective images from a prior IRB-approved study at University of Michigan where Y-90 PET/CT imaging was performed after Y-90 RE with glass microspheres for the purposes of lesion dosimetry [7]. From this larger data set, all patients that had a pre-treatment multiphase contrast-enhanced MRI and HCC lesions that appeared to be greater than approximately 2 mL in volume were selected. Nine patients with a total of 20 lesions were selected based on these criteria. MRI scans were performed on a 1.5 T GE Healthcare, 1.5 T Philips Healthcare, or a 3 T Siemens Healthineers scanner. In-plane resolution ranged from 0.69 to 1.37 mm, and slice thickness ranged from 2 to 3 mm. Acquisition and reconstruction protocols varied because some scans were obtained from outside hospitals.

### Contouring protocol

**Fig. 1:**
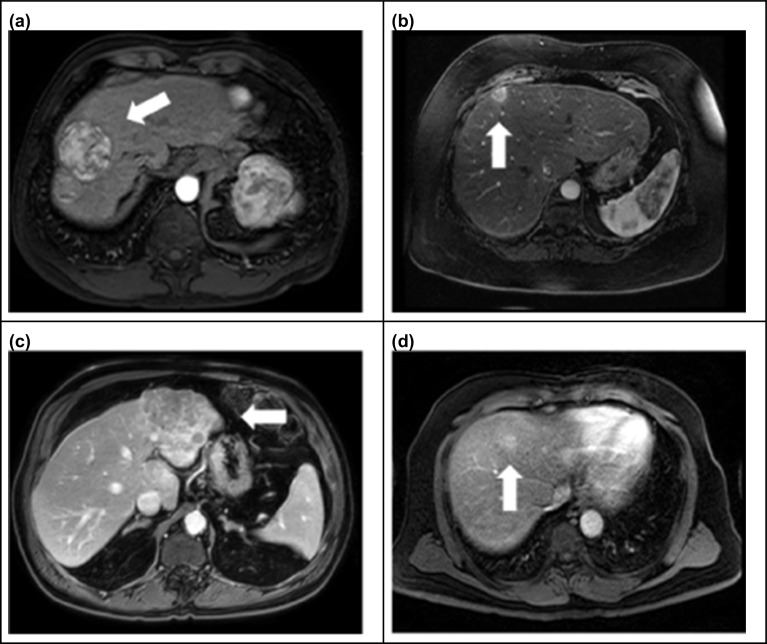
Example HCC lesions as seen on dynamic post-contrast T1-weighted fat-saturated MRI images. **a** Large, well defined (code 46.1, corresponding to patient #46, lesion #1). **b** Small, well defined (39.3). **c** Large, poorly defined (49.1). **d** Small, poorly defined (55.4). Each image is displayed in the optimum visualization window and phase used for segmentation

Three board certified, subspecialty trained abdominal radiologists with 20, 7, and 8 years of experience, respectively, were asked to contour the 20 lesions manually on baseline dynamic post-contrast T1-weighted fat-saturated MR images in the phase of contrast enhancement that maximized lesion visualization. Two of the radiologists (A and B) were asked to re-contour the same lesions at two additional rounds separated by 1-month intervals to assess intra-observer variability. This design gave a total of 140 observations—seven reads per lesion. At each round, the radiologists also recorded the longest lesion diameter in the axial plane according to RECIST criteria [16]. Each radiologist was provided a PowerPoint file with images indicating the general location of the lesions (for example, Fig. 1) and which phase to contour on. They were instructed to include necrotic and hypo-enhancing regions within the segmented volume as is standard in our lesion dosimetry protocol [7]. Lesion outlines were not specifically identified to minimize bias and Y-90 PET/CT images were not provided. All contouring and measuring were performed on MIMcloud version 7.1.3 (MIM Software, Cleveland). Radiologists were free to use any of the available tools with the software, but were encouraged to contour on the axial slices because of the higher resolution. The radiologists saved their data to a common cloud location and were not able to access data from any previous sessions.

The most experienced radiologist (A) classified lesion boundaries as well-defined or poorly-defined. Well-defined lesions were those which showed enhancement on the arterial phase and high contrast to the background liver parenchyma. Lesions were further classified as small or large, with small lesions defined as those with a mean volume of 8 mL or less across all reads.

### Dose metrics

Y-90 dose maps were available from our prior study [7]. Generation of patient-specific dose maps using the in-house developed Dose Planning Method (DPM) Monte Carlo (MC) code is described in detail in that study and is briefly summarized here. The inputs to DPM were the patient’s CT-derived density map and the quantitative Y-90 PET image acquired on a Siemens Biograph mCT with time of flight and reconstructed with 21 iterations, 1 subset of 3D-OSEM with resolution recovery and a 5-mm Gaussian post-filter. The output was the voxel-level dose rate map, which was converted to a dose map assuming physical decay only.

The baseline MRI was registered to the Y-90 PET/CT-derived dose map, and the lesion contours were transferred. Visually, if the automatic rigid registration was deemed unacceptable, manual fine-tuning of the alignment was performed using PET intensity as a guide, which is the process we use in our clinical dosimetry studies. The registration was performed once for each case and saved so that contours of subsequent rounds could be imported without having to re-register the images.

Dosimetry metrics were recorded for each of the 140 lesion contours transferred to the dose maps: mean absorbed dose without PVC, D10, and D90. The D10 and D90 represent the minimum dose, in Gy, delivered to 10% and 90% of the target volume, respectively. Additionally, a mean value PVC to correct for resolution effects was applied to the mean absorbed dose by scaling the value by a volume-dependent RC. The RC versus volume relationship, RC = − 0.934*v^−0.573^ + 0.883, used for this correction came from a previously reported phantom experiment using multiple spheres filled with known activity [7]. Because this is a mean value correction, RCs were not applied when reporting voxel-level metrics. Ideally, a voxel-level PVC should be applied to the image to improve accuracy of voxel dosimetry metrics, but such a correction is methodologically challenging, and there is not yet a well-validated practical method of doing so.

### Inter- and intra-observer variability

We started by comparing variability among contours drawn by the same radiologist (intra-observer) to variability among contours drawn by two different radiologists (inter-observer). The Dice coefficient is a measure of spatial overlap between two contours. Inter-observer Dice coefficients were calculated for each pair of reads executed by different radiologists on the same tumor during the same round, and intra-observer Dice coefficients were calculated for each pair of reads executed by the same radiologist on the same tumor across rounds. We expected inter-observer variability to exceed intra-observer variability; that is, contours drawn by the same radiologist should be more similar to each other than contours drawn by different radiologists. We verified this assumption using a two-sample *t*-test comparing the mean inter-observer Dice coefficient to the mean intra-observer Dice coefficient.

To evaluate sources of variance in repeated measurements, we fit a two-factor random effects models for repeated measures of volume, mean absorbed dose (both with and without RCs applied), and RECIST diameter measurements. Lesion and reader terms were treated as random effects (as opposed to fixed effects) because we take them to be randomly selected from a larger population of interest. That is, the specific readers in this study were not of primary importance; rather, the target of inference was the set of all possible clinicians who may contour HCC lesions. The same is true for the selected lesions themselves. This modeling strategy partitions overall variability in measurements into three sources: differences among the lesions themselves, differences between observers (inter-observer error), and differences within observers (intra-observer error). Each source of variability is assumed to have mean zero and an associated variance: *σ*^2^_lesion_, *σ*^2^_inter_, and *σ*^2^_intra_, due to lesion, inter-observer, and intra-observer differences, respectively. These three components sum to the total variance, *σ*^2^_total_. Models were fit for the whole set of observations, as well as for subsets by size and boundary definition. Subsets jointly defined by size and boundary definition could not be analyzed due to small sample size.

To assess the reliability of the measurements made by the three radiologists, inter- and intra-observer reliability coefficients were calculated [12, 17]. Such reliability coefficients are a form of intraclass correlation coefficients (ICCs) consistent with the random effects model defined above. The inter-observer reliability coefficient describes the consistency of measurements between readers and is expressed as:

$$\rho_{{{\text{inter}}}} = \frac{{\sigma_{{{\text{lesion}}}}^{2} }}{{\sigma_{{{\text{total}}}}^{2} }} = 1 - \frac{{\sigma_{{{\text{inter}}}}^{2} + \sigma_{{{\text{intra}}}}^{2} }}{{\sigma_{{{\text{total}}}}^{2} }}$$

A value closer to 1 indicates that the readers are more interchangeable; that is, more of the variability is attributable to the lesion and not the readers.

The intra-observer reliability coefficient describes the consistency and reproducibility of measurements within a single reader and is expressed as.:

$$\rho_{{{\text{intra}}}} = \frac{{\sigma_{{{\text{lesion}}}}^{2} + \sigma_{{{\text{inter}}}}^{2} }}{{\sigma_{{{\text{total}}}}^{2} }} = 1 - \frac{{\sigma_{{{\text{intra}}}}^{2} }}{{\sigma_{{{\text{total}}}}^{2} }}$$

A value closer to 1 indicates that an increased portion of the total variance is due to differences between lesions and differences between observers; that is, less variance in the outcome is due to random error within one observer.

### Uncertainty as a function of tumor volume

To understand how tumor volume impacts uncertainty in volume and mean absorbed dose, we fit models regressing relative uncertainty on contour volume, *v*. Uncertainty was measured by the coefficient of variation (CV), defined by the SD across the seven reads of the same lesion, scaled by the mean of those reads. Spearman correlation coefficients between *v* and CV are reported. Initial graphs indicated that a linear model would not be an appropriate fit for the data, and power models of the form ${\text{CV }} = \alpha v^{\beta }$ were found to be a good fit based on examination of residuals. Formal tests for the null hypothesis that *β* = 0 were conducted for each model to assess the fit.

### TCP example

We extended our study to determine the extent to which uncertainty in manual lesion contouring propagates to uncertainty in absorbed dose and probability of tumor control for an example data set. Volume and mean absorbed dose measurements for 89 lesions (from 28 patients with primary and secondary hepatic malignancies) treated with Y-90 radioembolization were obtained from a prior study by Dewaraja et al. [7], where tumor control probability (TCP) models were developed for these lesions. Models used a logit link, with Y-90 PET/CT-based mean absorbed dose (with RCs applied for PVC) as the covariate and binary tumor-level response classification at first follow-up, defined by lesion shrinkage criteria, as the outcome. The prior study did not include any uncertainty estimates. For each of the 89 lesions, we applied mean dose uncertainty (computed from the power model developed in the present study) to determine its effect on TCP using the following steps:

1. Utilizing the volume versus variability in mean dose function fitted to our original 20 lesions, predict the relative mean dose uncertainty for the given lesion based on its contour volume. Scale by measured mean dose to obtain expected standard deviation (SD). This value represents the SD we would expect to see for this lesion, given inter- and intra-observer uncertainty in volume contouring.
2. Compute measured mean dose ± 2 SD to get a plausible range of values for mean dose for this lesion, accounting for uncertainty.
3. Plug mean dose ± 2 SD into the previously derived TCP model (logit function).
4. Compute Δ_TCP_ = TCP(mean dose + 2SD)—TCP(mean dose—2SD). This quantity represents the plausible range of TCP values we would expect to see for this lesion, given the uncertainty in volume contour.

All analyses were performed using R version 4.1.1.

## Results

### Descriptive statistics and overall uncertainty

**Fig. 2:**
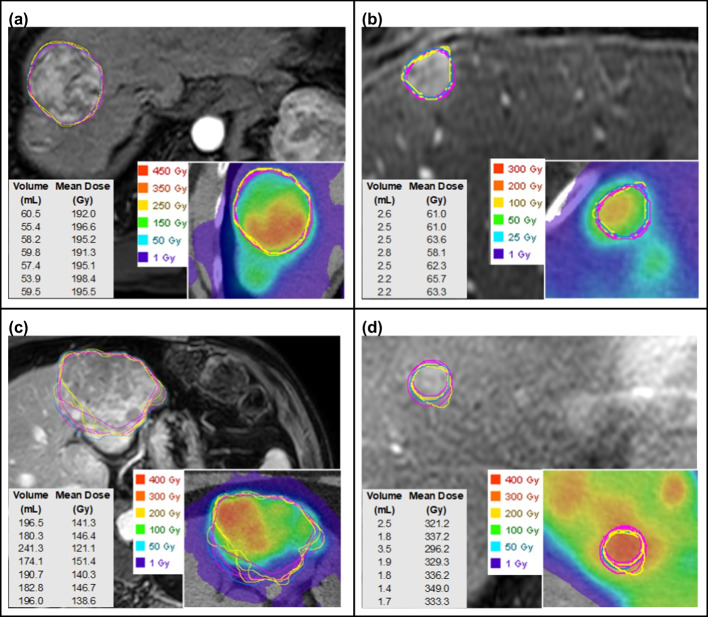
Radiologist-defined tumor contours on MRI corresponding to the four example lesions depicted in Fig. 1. Inserts show contours transferred to co-registered Y-90 PET/CT-based dose maps. Mean dose values are indicated before PVC. **a** Large, well defined (code 46.1). **b** Small, well defined (39.3). **c** Large, poorly defined (49.1). **d** Small, poorly defined (55.4). Contours drawn by the same radiologist are indicated in the same color (Pink = radiologist A, Yellow = radiologist B, Blue = radiologist C)

**Fig. 3:**
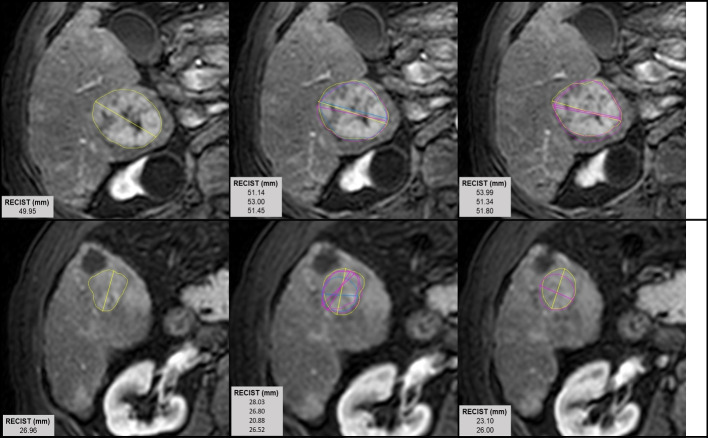
Radiologist-defined RECIST measurements on two example lesions: Lesion codes 19.2 (top row) and 19.3 (bottom row). Radiologists were free to choose the MRI slice on which they indicated the diameter. Within each row, each of the three images depicts a different MRI slice. Diameters drawn by the same radiologist are indicated in the same color (Pink = radiologist A, Yellow = radiologist B, Blue = radiologist C). Lesion contours are included for context. Each image is displayed in the optimum visualization window and phase used for segmentation

Example contours are shown in Fig. 2, and example (longest) diameters according to RECIST criteria are shown in Fig. 3. Individual values of lesion volume, mean absorbed dose, and diameters corresponding to each reader and round are plotted in Additional file 1: Figures S1–S4. Table 1 displays descriptive statistics for lesion measurements and absorbed dose metrics by lesion aggregated across all seven repeated contours. The lesions selected for this study covered a wide range in volume, with average lesion volume ranging from 1.8 to 194.5 mL (interquartile range 3.7–33.6 mL). Nine lesions were classified as large and 11 small; the most experienced participating radiologist deemed 12 lesions to have well-defined margins and 8 to have poorly-defined margins. Mean absorbed dose without PVC ranged from 15.0 to 468.9 Gy (interquartile range 63.8 to 245.6 Gy). Mean absorbed dose with RCs applied ranged from 23.5 to 1629.0 Gy (interquartile range 142.3–441.7 Gy).

**Table 1:** Descriptive statistics by lesion, summarized by mean (SD) across the 7 reads

Lesion code	Well defined?	Size classification	Volume (mL)	Mean absorbed dose (Gy)	Mean absorbed dose with RC (Gy)	RECIST (mm)	D10 (Gy)	D90 (Gy)
*Patient 19*								
19.2	Yes	Large	54.6 (2.9)	131.8 (3.8)	167.1 (5.2)	51.8 (1.3)	196.7 (1.9)	64.6 (5.5)
19.3	No	Small	6.1 (1.1)	99.6 (6.5)	183.0 (22.5)	25.5 (2.5)	161.2 (5.5)	52.3 (3.7)
19.4	No	Small	5.1 (1.5)	47.9 (1.4)	98.6 (20.0)	23.0 (2.9)	61.6 (2.7)	35.1 (1.9)
19.5	Yes	Small	3.0 (0.6)	52.5 (3.1)	144.5 (34.7)	20.9 (2.8)	70.3 (1.8)	34.5 (4.0)
*Patient 39*								
39.1	Yes	Small	1.8 (0.3)	159.6 (2.7)	819.4 (280.8)	18.3 (1.3)	205.8 (1.7)	109.8 (5.2)
39.3	Yes	Small	2.5 (0.2)	62.1 (2.4)	193.0 (23.9)	18.5 (0.6)	110.4 (2.0)	22.5 (2.6)
*Patient 46*								
46.1	Yes	Large	57.8 (2.4)	194.9 (2.5)	246.0 (3.7)	54.6 (1.8)	351.2 (1.3)	54.9 (3.7)
46.2	Yes	Small	6.2 (0.8)	74.4 (2.7)	135.5 (12.1)	29.0 (1.6)	104.9 (1.7)	44.2 (3.1)
*Patient 49*								
49.1	No	Large	194.5 (22.2)	140.8 (9.8)	168.1 (12.2)	103.4 (5.1)	284.0 (7.8)	26.5 (10.4)
49.2	Yes	Small	3.9 (0.9)	168.8 (8.3)	383.4 (64.6)	22.1 (1.2)	238.0 (6.8)	96.5 (10.1)
*Patient 55*								
55.1	No	Large	26.6 (5.1)	64.3 (8.4)	87.0 (11.0)	45.7 (6.2)	145.0 (5.3)	4.0 (3.3)
55.2	Yes	Large	24.6 (3.3)	49.8 (1.3)	68.0 (2.6)	41.3 (1.4)	75.4 (1.3)	23.2 (1.5)
55.3	Yes	Large	10.4 (0.9)	15.0 (1.4)	23.5 (2.5)	37.0 (2.2)	34.2 (1.5)	2.3 (0.6)
55.4	No	Small	2.1 (0.7)	328.9 (16.7)	1629.0 (899.0)	17.3 (1.7)	394.4 (9.7)	264.1 (23.3)
*Patient 61*								
61.1	No	Large	9.8 (1.9)	164.4 (7.7)	263.9 (26.6)	32.9 (2.0)	245.2 (4.2)	83.0 (8.8)
*Patient 62*								
62.1	Yes	Large	58.5 (11.5)	425.5 (27.9)	538.5 (44.6)	57.0 (9.8)	727.2 (25.5)	153.4 (18.4)
*Patient 69*								
69.1	No	Small	6.8 (1.9)	312.2 (27.5)	566.8 (115.4)	28.0 (1.2)	616.3 (38.9)	93.1 (12.5)
69.2	No	Small	3.0 (0.7)	468.9 (31.7)	1,284.6 (324.3)	20.7 (1.9)	699.9 (24.5)	260.2 (34.1)
69.3	Yes	Small	7.4 (1.0)	223.4 (8.5)	383.4 (32.3)	24.8 (2.2)	325.4 (7.2)	138.3 (10.9)
*Patient 74*								
74.1	Yes	Large	125.4 (20.2)	337.3 (22.7)	409.4 (29.9)	86.5 (7.2)	578.8 (17.3)	105.1 (19.9)

There was a considerable range of uncertainty (CV% across seven measurements) in volume, ranging from 4.2 to 34.7% with a mean of 17.2%. Uncertainty in RECIST diameter measurement within a given lesion was lower than that of contour volume. The uncertainty in diameter measurements ranged from 2.6 to 17.2%, with a mean of 7.7%. Uncertainty in mean absorbed dose varied from 1.3 to 13.1% with a mean of 5.4% before PVC and 1.5 to 55.2% with a mean of 15.1% when RCs were applied. Regarding dose–volume histogram metrics, uncertainty in D10 ranged from 0.4 to 6.3% with a mean of 2.7%. Variability in D90 was much higher, with CV% ranging from 4.7 to 83.1%, with a mean of 15.7%.

### Inter- and intra-observer variability

Dice coefficient values for the contours had a left skewed distribution and ranged from 0.48 to 0.95 (mean = 0.82, median = 0.84). Mean inter-observer Dice coefficient was 0.79 (SE = 0.009), while mean intra-observer Dice coefficient was 0.85 (SE = 0.006). Histograms of Dice coefficients are plotted in Additional file 1: Figure S5. A two-sample t-test confirmed that the mean Dice coefficient was significantly higher for pairs of contours traced by the same reader than for pairs of contours traced by different readers (*T* = − 4.80, *p* < 0.001).

Table 2 presents the percent of variance attributable to inter- and intra-observer components (after accounting for variance between lesions) for volume, mean absorbed dose with RC, mean dose without RC, and RECIST diameter. Corresponding intra- and inter-observer reliability ICCs are presented in Table 3.

**Table 2:** Percentage of variance attributable to inter- and intra-observer differences for volume, mean absorbed dose, and RECIST diameter measurements in all lesions and covariate-defined subgroups after accounting for inherent variability due to the lesions

Component of variance	All lesions (*n* = 140) (%)	Small lesions (*n* = 77) (%)	Large lesions (*n* = 63) (%)	Well-defined lesions (*n* = 84) (%)	Poorly-defined lesions (*n* = 56) (%)
*Volume*					
Inter-observer	75.8	60.9	76.1	61.4	88.4
Intra-observer	24.2	39.1	23.9	38.6	11.5
*Mean absorbed dose (without RC)*					
Inter-observer	61.5	54.5	76.9	75.0	53.3
Intra-observer	38.5	45.5	23.1	25.0	46.7
*Mean absorbed dose (with RC)*					
Inter-observer	26.0	25.9	65.0	70.7	21.9
Intra-observer	74.0	74.1	35.0	29.3	78.1
*RECIST diameter*					
Inter-observer	73.5	61.6	75.4	88.7	31.3
Intra-observer	26.5	38.4	24.6	11.3	68.8

**Table 3:** Inter- and intra-observer reliability ICCs for all outcomes

	All lesions (*n* = 140)	Small lesions (*n* = 77)	Large lesions (*n* = 63)	Well-defined lesions (*n* = 84)	Poorly defined lesions (*n* = 56)
*Volume*					
*ρ*_intra_	0.992	0.878	0.989	0.983	0.997
*ρ*_inter_	0.967	0.735	0.954	0.956	0.974
*Mean dose (without RC)*					
*ρ*_intra_	0.995	0.995	0.997	0.997	0.993
*ρ*_inter_	0.987	0.988	0.987	0.989	0.986
*Mean dose (with RC)*					
ρ_intra_	0.818	0.788	0.993	0.957	0.790
ρ_inter_	0.754	0.714	0.980	0.853	0.732
*RECIST diameter*					
*ρ*_intra_	0.991	0.898	0.985	0.994	0.989
*ρ*_inter_	0.966	0.743	0.939	0.947	0.984

*ρ*_intra_ = intra-observer reliability coefficient; *ρ*_inter_ = inter-observer reliability coefficient

### Uncertainty as a function of tumor volume

**Fig. 4:**
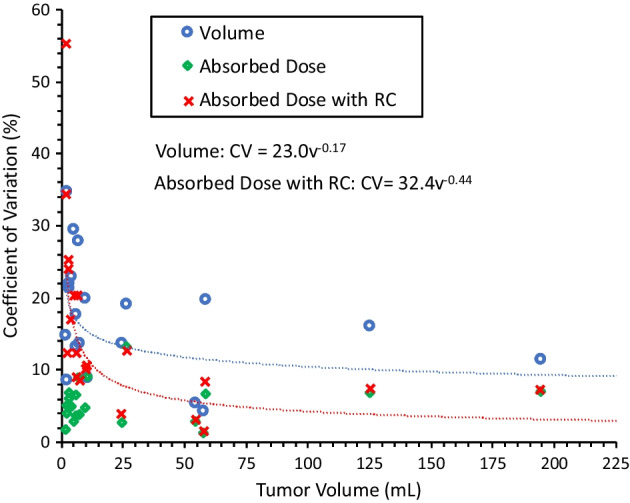
Volume uncertainty and mean absorbed dose uncertainty (with and without PVC) plotted as a function of volume

For each lesion, uncertainty in volume and mean absorbed dose with and without PVC are plotted versus lesion volume in Fig. 4. Volume and volume uncertainty were modestly correlated (*ρ*_Spearman_ = − 0.44); volume and dose (with PVC) uncertainty were strongly correlated (*ρ*_Spearman_ = − 0.84); volume and dose (without PVC) uncertainty were not correlated. The data provide sufficient evidence that *β* ≠ 0 (exponent coefficient) in both models (*p* = 0.0497 for volume uncertainty; *p* = 0.0002 for dose (with PVC) uncertainty). The functional forms of the fitted curves are displayed in Fig. 4. For large (> 8 mL) lesions, the uncertainty in volume was on average 13.1% (4.2–19.9%) and for mean absorbed dose with PVC was on average 7.2% (1.5–12.6%). For small lesions, the uncertainty in volume was on average 20.6% (8.5–34.7%) and for mean absorbed dose with PVC was on average 21.7% (8.4–55.2%).

### TCP example

**Fig. 5:**
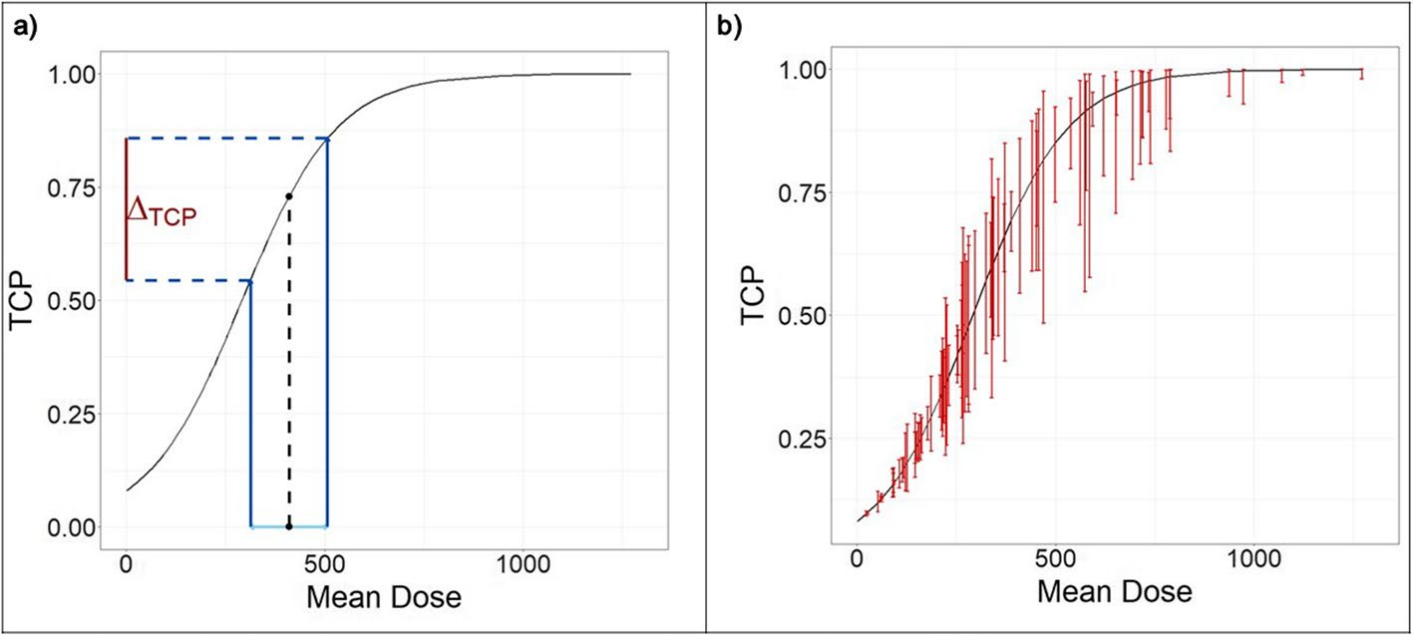
Volume uncertainty propagated into tumor control probability (TCP), applied on the data and TCP curve fitted in the study by Dewaraja et al. [7]. **a** Procedure for computing Δ_TCP_ for an example patient. **b** Original TCP curve (black line) overlaid by Δ_TCP_ for 89 liver lesions

Figure 5 illustrates the propagation of volume and mean dose uncertainty into TCP. Panel A presents the procedure for finding Δ_TCP_ for an example patient, represented by the red bar along the y-axis. Repeating the same procedure, we overlay the Δ_TCP_ values for all 89 lesions in Panel B. Recall that the length of the bars is determined by the volume/uncertainty relationship; thus, even two lesions with similar mean dose can have error bars of noticeably different sizes if their volumes differ. Among the lesions included, mean Δ_TCP_ was 16.2% and maximum Δ_TCP_ was 48.5%, with 27.0% of lesions having a TCP difference of at least 25% when accounting for standard uncertainty.

## Discussion

Variability in delineating the VOI is a primary source of uncertainty along the radionuclide therapy dosimetry chain [4, 13]. The degree of contrast enhancement, spatial resolution, and tumor volume are main factors that restrict the precision with which the observer can assess the lesion boundary on anatomical imaging modalities. Ideally, we would create an average VOI boundary across multiple observers, but this is an impractical use of resources in clinical practice. Our study simulated that ideal situation by having three radiologists repeatedly outlines the same tumors on a historical data set. Leveraging these repeated measurements, we have quantified how observer effects contribute to uncertainty in VOI delineation and the corresponding absorbed dose estimates. We have also provided a model that can be used in future studies to estimate uncertainty under similar imaging and segmentation conditions.

Dice coefficients revealed that the mean intra-observer spatial overlap (0.85) is significantly greater than the mean inter-observer overlap (0.79) in contours, which substantiates the assumption that operators tend to agree with themselves more than they agree with other operators. Although residual memory bias can be a confounding factor, we mitigated this effect by separating contouring sessions by 1 month. Based on our random effects models, once accounting for inherent differences between lesions, the majority of remaining variance in volume and RECIST diameter is attributable to inter-observer variability. However, this conclusion did not hold for RC-corrected mean absorbed dose overall, in small lesions, or in poorly defined lesions. Sensitivity analyses revealed this result to be largely attributable to one very small, poorly defined lesion (Fig. 2d, lesion code 55.4). Radiologist A defined Lesion 55.4 to have a volume of 1.8 mL on one read and 3.5 mL on another; this difference was further magnified by the PVC because the RC versus volume curve has a steep gradient at small volumes, thereby creating large variation within Radiologist A’s mean dose measurements. Sensitivity analysis excluding this outlier lesion shows that inter-observer variability is larger than intra-observer variability across all outcomes and subgroups, as expected (Additional file 1: Table S1).

The inter- and intra-observer reliability coefficients presented in Table 3 suggest substantial agreement both between and within readers. Most intra-observer reliabilities are greater than 0.9, reinforcing the conclusion that observations of the same case made by the same reader are generally consistent and reproducible. Encouragingly, inter-observer reliabilities are nearly all above 0.8, reflecting substantial agreement *between* readers, as well. These findings are consistent with the findings of Meyers et al. [14], who report an inter-observer ICC of 0.94 for volume and 0.73 for mean absorbed dose for delineation on contrast-enhanced CT in a similar cohort of HCC patients treated with Y-90 RE. Similarly, McErlean et al. [11] determined intra- and inter-observer reliabilities of 0.957 and 0.954, respectively, for RECIST measurements on CT images, which compare well with our values.

We provide fitted uncertainty curves that can potentially be applied to future patient studies to produce an informed estimate of standard uncertainty in tumor volume and mean absorbed dose. We expect that the findings will be also applicable to hepatic lesion types other than HCC, because lesion contouring was done on the contrast enhanced sequences of MRI, which is routinely used for evaluation of any primary or secondary hepatic malignancies. However, it is an important caveat that the fitted functions depend heavily on multiple factors including the contouring method, imaging modality/parameters, and the PVC method used. In general, uncertainty is reduced when progressing from volume to mean dose calculation (Fig. 4), because the dose maps are blurred out by motion and the limited spatial resolution of Y-90 PET. With PVC, the sharp rise in the mean dose uncertainty at small volumes is partly due to the sharp rise in the volume-dependent RC curve at small volumes. Although PVC increases the variability, it is well accepted that the accuracy of the mean dose estimate increases with this correction [18].

The relationship between volume and mean absorbed dose uncertainty ascertained by our empirical approach can be compared with results presented by Finnochiaro et al. [13], who used an analytical equation that captures uncertainty in volume as a function of image resolution. Although the trend is the same, our estimates of uncertainty are much lower. For example, at a volume of 100 mL, Fig. 4 estimates just over 10% uncertainty in volume and about 5% uncertainty in mean dose with PVC. In contrast, Finnochiaro et al. estimate over 30% uncertainty in volume and over 25% uncertainty in mean dose using phantom-based RCs for PVC as in our study. This difference can be mostly attributed to the fact that we used MRI for tumor segmentation, which is much higher resolution than the SPECT imaging used in the comparison study. Furthermore, although uncertainty in segmentation was the dominant factor, they included other sources of uncertainty from the dosimetry chain.

We applied our model of segmentation uncertainty from the present study to determine how it impacts a model for probability of tumor control published previously by our group. We found the largest impact on TCP among lesions with intermediate mean dose values, which is attributable to the shape of the logistic curve (Fig. 5). Overall, our analysis predicts that approximately one in four lesions would have Δ_TCP_ of at least 25% when accounting for standard uncertainty. Although TCP is not presently formally utilized as a clinical decision-making aide, it reflects expected treatment efficacy and patient outcomes. A clinician might make different treatment decisions given 50% probability of tumor control compared to 75% probability.

Our characterization of the segmentation uncertainty on absorbed dose reporting could be helpful in planning RE to reduce its contribution to treatment failures. One potential solution is to devise a more reproducible and repeatable segmentation method. Another potential solution worth investigating is to plan RE infusions with additional dose, forcing tumor absorbed doses deeper into the plateau region of a dose–response curve. Such increases in dose may be possible for radiation segmentectomies where there is minimal dose delivered to normal liver parenchyma, which also must be considered, in addition to other clinical factors specific to the patient. Nevertheless, demonstrated benefit of personalized dosimetry in a recent trial [3] suggests that such escalation is feasible in select cases.

We focused our evaluation to looking at variability as a function of volume, although factors such as the acquisition/quality of the MRI, lesion physiology-related factors (contrast enhancement relative to liver parenchyma), and shape of the lesions will also impact the manual segmentation. To reduce imaging-related factors, we chose images of similar quality: all were dynamic post-contrast T1-weighted fat-saturated MRI without large motion artifacts acquired on 1.5 Tesla or greater state-of-the-art systems. To reduce impact of physiology, the phase of post-contrast enhancement that showed the best lesion contrast was selected and lesions were contoured by all radiologists on this phase. Operators were instructed to include necrotic regions within the lesion contour as it is challenging to differentiate between true necrosis and hypo-enhancing regions (for example, Fig. 2c). Our analysis did not account for inherent volume uncertainty from the finite MRI voxel size, but this effect is considered small in comparison to the lesion size included in the study (2–194 mL). Furthermore, although volume uncertainty is expected to be the greatest contributor to dose uncertainty, there are other potential sources of uncertainty along the dosimetry chain [4, 5, 13] that we did not investigate. This includes misregistration between the baseline image used for segmentation and the PET/CT; however, this concern is reduced by our approach of manually fine tuning the transferred contour location, guided by the PET intensity. Although widely used in dosimetry applications, the uncertainty associated with using RCs derived from sphere measurements for patient tumors that vary not only in volume but also in shape, location and activity non-uniformity is well known [18]. Quantifying all effects contributing to absorbed dose uncertainty was beyond the scope of our study aims, but it motivates future work to integrate the analysis conducted in the current study with other sources of variation. Similarly, it is important to note that our analysis of change in TCP only captures uncertainty arising from VOI delineation and does not account for uncertainty associated with our prior analytical model for TCP. Hence, the reported variability should be considered as a lower limit. Future studies could also benefit from the inclusion of more than three radiologists but are expensive to implement. Additionally, in the absence of ground truth, our study was constrained to assessing observer variability and was not equipped to ascertain accuracy. A previous study of accuracy and reproducibility using synthetic brain MR images reported that manual tracers tend to overestimate lesion margins compared to automated techniques [19].

Reduction in inconsistencies among radiologists reduces variability in dosimetry and has potential to reduce variability in patient outcomes when using dosimetry-guided internal radionuclide therapy. To reduce operator variability, a standardized imaging and segmentation protocol should be used. Optimized MRI protocols include appropriately timed post-contrast imaging that enable better visualization of margins between lesions and normal liver parenchyma and multi-phase imaging provides the opportunity to pick the phase that is less effected by motion. Access to robust image analysis tools that includes optimized auto-windowing for image display is also of importance to reduce operator variability. Although we did not specify the zoom factor to use in the current study, having operators use a consistent zoom on the lesion is helpful to avoid losing some fine details on the borders when zoomed out too much compounding the inherent variability in the process. Furthermore, consensus should be reached by operators on segmentation strategy for dosimetry, such as whether to include or exclude necrotic regions from the lesion contour.

## Conclusion

In this multi-operator/session study, the uncertainty in segmented tumor volumes as measured by the CV ranged from 4.2 to 34.7% with an average value of 17.2% across the 20 HCC lesions. The corresponding uncertainty in Y-90 PET derived mean absorbed dose was lower (average 5.4%) due to blurring effects associated with motion and limited PET spatial resolution but increased with the use of volume-dependent RCs (average 15.1%). This is especially true for small (2–8 mL) lesions, where the CV in mean absorbed dose with PVC was 21.7% on average (range 8.4–55.2%).

## Supplementary Information

**Additional file1.** **Figure S1**: Dot plot of volume measurements grouped by lesion. Dots of the same color indicate measurements attributed to the same radiologist. **Figure S2**: Dot plot of mean absorbed dose measurements (without PVC) grouped by lesion. Dots of the same color indicate measurements attributed to the same radiologist. **Figure S3**: Dot plot of mean absorbed dose measurements (with RC applied) grouped by lesion. Dots of the same color indicate measurements attributed to the same radiologist. **Figure S4**: Dot plot of RECIST diameter grouped by lesion. Dots of the same color indicate measurements attributed to the same radiologist. **Figure S5**: Overlapping histograms of intra- and inter-observer Dice coefficients. Higher values indicate greater overlap between contours. **Table S1**: Percentage of variance attributable to inter- and intra-observer differences for mean absorbed dose when excluding one outlier lesion (55.4), after accounting for inherent variability due to the lesions.

## Data Availability

Data can be made available after contacting the authors.
