# Supplementary material for: Intra- and inter-operator variability in MRI-based manual segmentation of HCC lesions and its impact on dosimetry
Source: EJNMMI Phys. 2022 Dec 21;9:90. doi: 10.1186/s40658-022-00515-6 (PMC9772368; doi:10.1186/s40658-022-00515-6)
Supplement: Supplementary file 1 — Additional file1. Figure S1: Dot plot of volume measurements grouped by lesion. Dots of the same color indicate measurements attributed to the same radiologist. Figure S2: Dot plot of mean absorbed dose measurements (without PVC) grouped by lesion. Dots of the same color indicate measurements attributed to the same radiologist. Figure S3: Dot plot of mean absorbed dose measurements (with RC applied) grouped by lesion. Dots of the same color indicate measurements attributed to the same radiologist. Figure S4: Dot plot of RECIST diameter grouped by lesion. Dots of the same color indicate measurements attributed to the same radiologist. Figure S5: Overlapping histograms of intra- and inter-observer Dice coefficients. Higher values indicate greater overlap between contours. Table S1: Percentage of variance attributable to inter- and intra-observer differences for mean absorbed dose when excluding one outlier lesion (55.4), after accounting for inherent variability due to the lesions. [file 40658_2022_515_MOESM1_ESM.docx]

**Intra- and Inter-operator variability in MRI-based manual segmentation of HCC lesions and its impact on dosimetry**

**Authors: Authors:** Elise C. Covert,^1^ Kellen Fitzpatrick,^2^ Justin Mikell,^3^ Ravi K. Kaza,^4^ John D. Millet,^2^ Daniel Barkmeier,^2^ Joseph Gemmete,^2^ Jared Christensen,^2^ Matthew J. Schipper,^1,3^ Yuni K. Dewaraja^2^

**Supplemental Data**


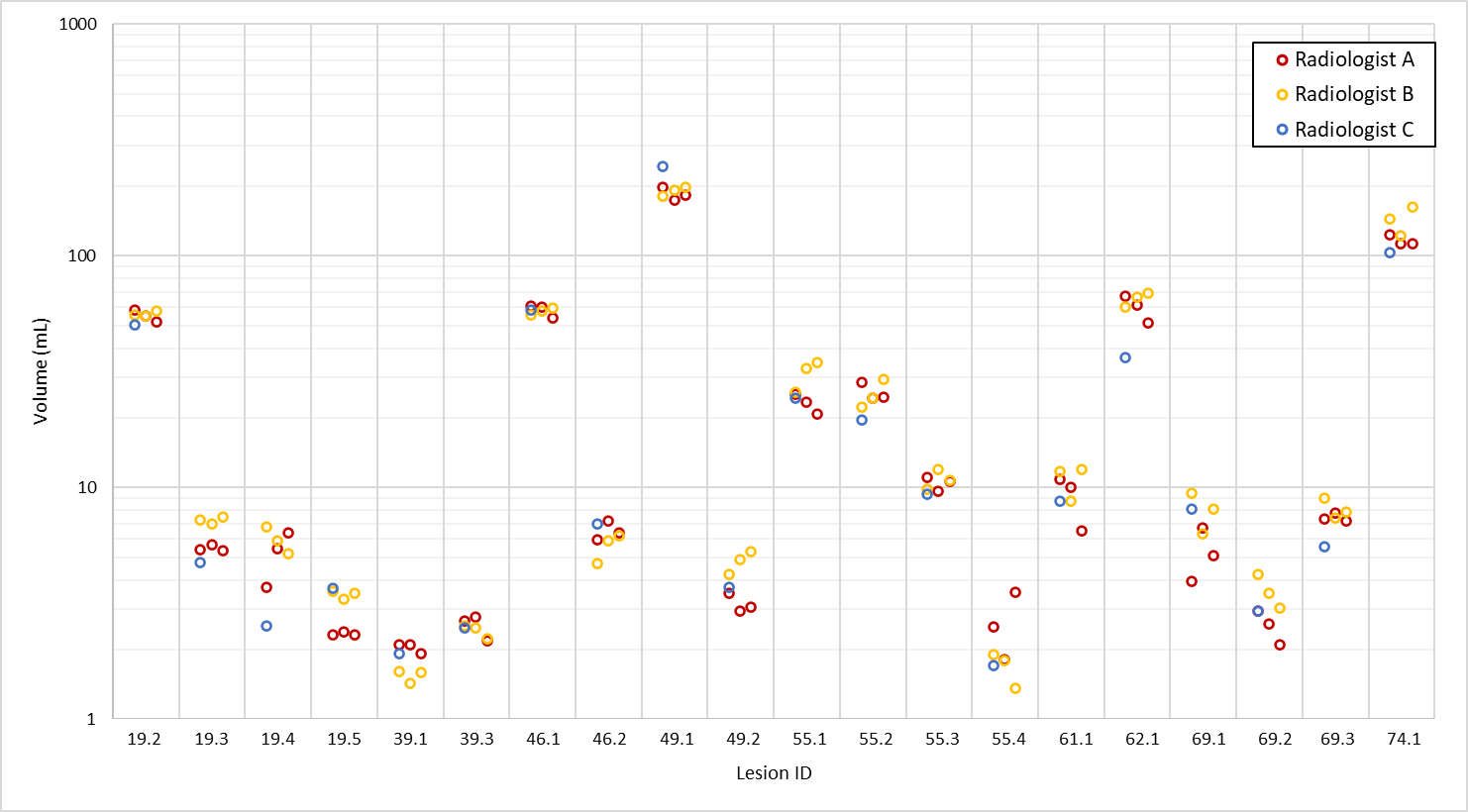


**Fig. S1** Dot plot of volume measurements grouped by lesion. Dots of the same color indicate measurements attributed to the same radiologist.

**Fig. S2** Dot plot of mean absorbed dose measurements (without PVC) grouped by lesion. Dots of the same color indicate measurements attributed to the same radiologist.


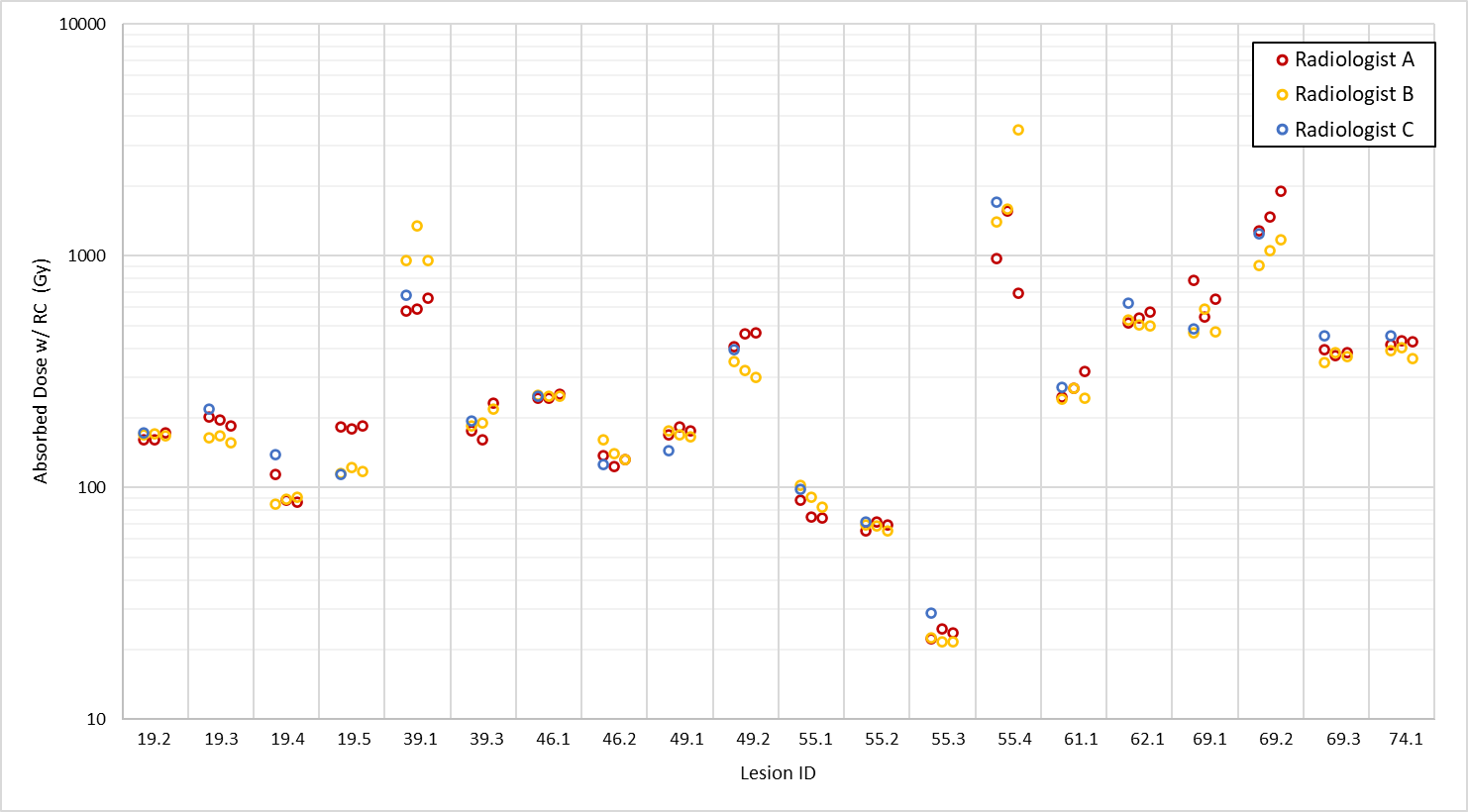


**Fig. S3** Dot plot of mean absorbed dose measurements (with RC applied) grouped by lesion. Dots of the same color indicate measurements attributed to the same radiologist.


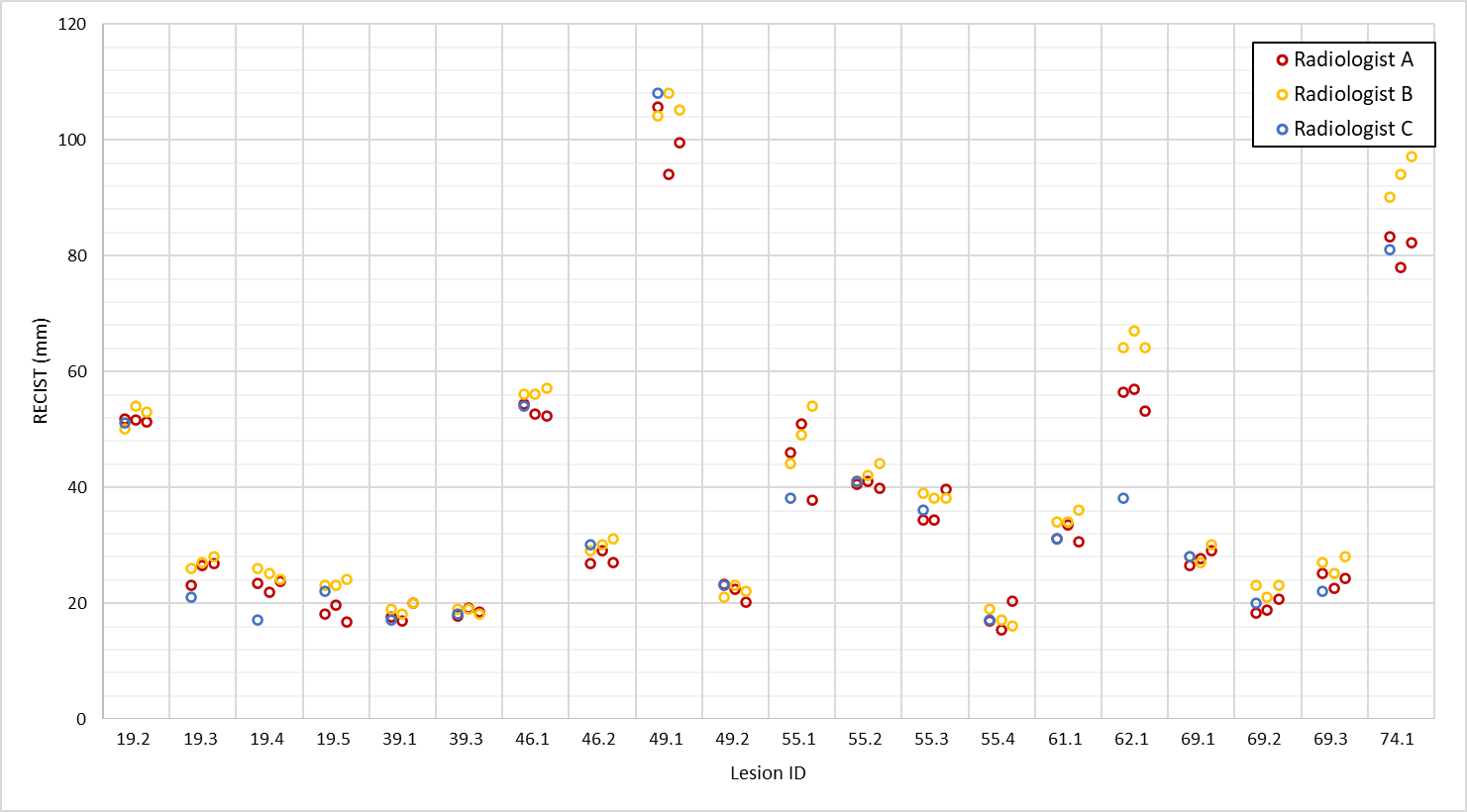


**Fig. S4.** Dot plot of RECIST diameter grouped by lesion. Dots of the same color indicate measurements attributed to the same radiologist.


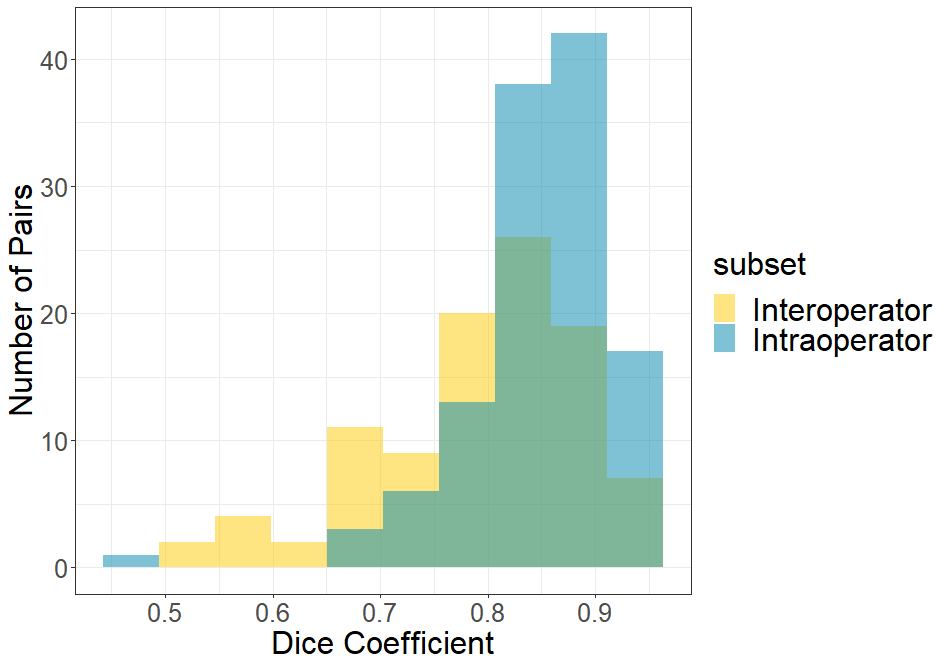


**Fig. S5** Overlapping histograms of intra- and inter-observer Dice coefficients. Higher values indicate greater overlap between contours.

**Table S1** Percentage of variance attributable to inter- and intra-observer differences for mean absorbed dose when excluding one outlier lesion (55.4), after accounting for inherent variability due to the lesions.

| **Component of Variance** | **All Lesions (n = 133)** | **Small Lesions**  **(n = 70)** | **Large Lesions**  **(n = 63)** | **Well-Defined Lesions**  **(n = 84)** | **Poorly-Defined Lesions**  **(n = 49)** |
| --- | --- | --- | --- | --- | --- |
| **Volume** |  |  |  |  |  |
| Inter-observer | 76.5% | 62.7% | 76.1% | 61.4% | 88.5% |
| Intra-observer | 23.5% | 37.3% | 23.9% | 38.6% | 11.5% |
| **Mean Absorbed Dose (without RC)** |  |  |  |  |  |
| Inter-observer | 69.2% | 58.3% | 76.9% | 75.0% | 57.1% |
| Intra-observer | 30.8% | 41.7% | 23.1% | 25.0% | 42.9% |
| **Mean Absorbed Dose (with RC)** |  |  |  |  |  |
| Inter-observer | 57.6% | 57.7% | 65.0% | 70.7% | 48.9% |
| Intra-observer | 42.4% | 42.3% | 35.0% | 29.3% | 51.1% |
